# Josephson Currents and Gap Enhancement in Graph Arrays of Superconductive Islands

**DOI:** 10.3390/e23070811

**Published:** 2021-06-25

**Authors:** Massimiliano Lucci, Davide Cassi, Vittorio Merlo, Roberto Russo, Gaetano Salina, Matteo Cirillo

**Affiliations:** 1 Dipartimento di Fisica and *MINAS* Lab, Università di Roma “Tor Vergata”, 00133 Roma, Italy; massimiliano.lucci@roma2.infn.it (M.L.); merlo@roma2.infn.it (V.M.); 2 Istituto Nazionale di Fisica Nucleare, Sezione di Roma “Tor Vergata”, 00133 Roma, Italy; salina@roma2.infn.it; 3 Dipartimento di Scienze Matematiche, Fisiche ed Informatiche, Università di Parma, 43124 Parma, Italy; davide.cassi@unipr.it; 4 Consiglio Nazionale delle Ricerche, CNR-ISASI, Via Castellino 111, 80131 Napoli, Italy; roberto.russo@na.isasi.cnr.it

**Keywords:** phase transitions, superconductivity, superconductive tunnelling, arrays of Josephson junctions

## Abstract

Evidence is reported that topological effects in graph-shaped arrays of superconducting islands can condition superconducting energy gap and transition temperature. The carriers giving rise to the new phase are couples of electrons (Cooper pairs) which, in the superconducting state, behave as predicted for bosons in our structures. The presented results have been obtained both on star and double comb-shaped arrays and the coupling between the islands is provided by Josephson junctions whose potential can be tuned by external magnetic field or temperature. Our peculiar technique for probing distribution on the islands is such that the hopping of bosons between the different islands occurs because their thermal energy is of the same order of the Josephson coupling energy between the islands. Both for star and double comb graph topologies the results are in qualitative and quantitative agreement with theoretical predictions.

## 1. Introduction

Topological matter represents an expanding field in condensed matter physics and books and reviews on the subject have appeared describing several aspects of the topic, demonstrating the interest from the fundamental and applied physics points of view [1,2]. The Nobel prize awarded in 2016 to David J. Thouless, F. Duncan M. Haldane, and J. Michael Kosterlitz for theoretical discoveries of topological phase transitions and topological phases of matter [3] was recognition of the efforts devoted to investigations in a research field which has intrigued the international community of condensed matter and solid state physics in the last five decades.

Superfluids, since the work of London [4,5], have represented an example that matter can become organized in terms of long range quantum coherence, now also addressed as topological order, an order stable even in spite of local, microscopic deformations [6]. Indeed, superfluid transitions are examples of transitions that have limited relation with structural symmetries but do abruptly modify transport thermodynamics properties due to the breaking of symmetry between macroscopic wave functions and energy. Flux quantization in superconductors and vortices in superfluid helium represent striking evidence of long range coherence of the wave functions in superfluids [7].

The experiments on ultracold alkali elements vapors (leading to the 2001 Nobel prize in physics) showing that a Bose–Einstein condensation (BEC) for atomic systems exists [8,9], activated a strong input of interest toward phenomena generated by long range coherence in physical systems. In spite of the substantial “structural” difference with superconducting and superfluid condensates, and in spite of the fact that the onset of superfluidity and superconductivity cannot be accounted for by Bose–Einstein condensation, one must say that most of the effects predicted by London [4,5] for the long range order of wave functions (observed in superfluidity and superconductivity) have been measured; even in alkali gas condensates for which spectacular evidence of flux quantization has been experimentally observed in a rotating bucket experiment of the gas vapors, similar to that performed with rotating superfluid helium [10].

The wave of interest generated by the BEC in alkali vapors led a group of theorists to investigate “inhomogeneous”, graph-shaped structures which could display specific evidence of “macroscopic order” [11,12]. The proposed physical systems could be either ultracold atomic systems or arrays of superconductive islands connected by specific potentials. The superconductive arrays are a discrete 2D system in which a BEC transition could be triggered, not by confining potentials but by specific topological inhomogeneities which could have an effect on boson’s (Cooper pairs) condensation [11,12,13]. The subject attracted interest even from groups of mathematicians since the technical evidence of the transition had to do with the hidden spectrum of operators [14]. As far as superconductivity is concerned, the predicted “topological” BEC transition could occur only below the superconducting condensation temperature when the electrons would form Cooper pairs with net zero momentum, a condition analogous to that necessary for observing Berezinskii–Kosterlitz–Thouless transitions in planar superconductive arrays [15,16,17].

**Figure 1:**
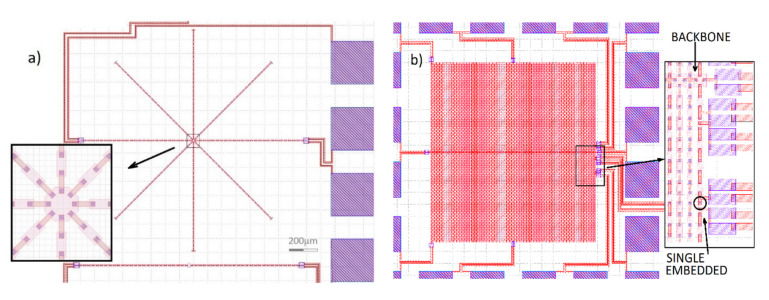
(**a**) Layout of an eight-rays star array chip also showing an enlargement of the central island; The “linear” array at the bottom of the layout is the reference array; The inset shows the central island of the star array having a volume which is eight times that of the single islands; (**b**) CAD layout of a double comb structure: the “backbone” is along the central horizontal array, whereas each of the one hundred vertical array is a “finger” array. The “central” finger array is the one located at the center while the “lateral” finger arrays are those located at the left and right extremities. The inset shows the portion of lateral (right) finger array where contacts were available to test single junctions embedded in the lateral finger array. The single junction that was tested is circled; for clarity, the position of the backbone array is also indicated in the zoom.

Several experiments have been performed in which evidence of boson distribution gradients in planar arrays, with specific topological structures, has been provided [18,19,20,21]. In these experiments the distribution of bosons on the islands was probed measuring current-voltage characteristics of series arrays of the Josephson junctions originated by oxide barriers between the superconductive islands; the junctions were also providing the coupling potential. The main strategy of these experiments relied on comparisons between series array embedded in the graph network being considered, and “sentinel” arrays, namely geometrically identical arrays, being placed on the same chip and having the same geometry and current density of the embedded structure, as shown in Figure 1a for a “star” graph. This position of the problem allows discriminating effects that are solely due to the specific embedding topology. In the present paper we report on results obtained on arrays of star geometry and arrays of double comb geometry. The CAD design of our double comb structures is shown in Figure 1b; here we also show a magnification of the portion of lateral finger where a single junction of a finger array can be contacted. Both arrays of double comb geometry [18,19,21] and arrays of star geometry [20] have been subject to both theoretical and experimental investigations.

## 2. Results and Discussion

In Figure 1a we see the CAD layout of a typical star array geometry consisting of superconductive islands connected by Josephson junctions; the array we see below the star structure at the bottom is the “reference” array having the same geometrical shape of the central array of the star. The central island of the star array has a volume which is eight times that of the single islands, in order to have uniform charge carriers density all over the islands. The samples we designed for the experiments were fabricated using niobium technology, based on Nb-NbAlOx-Nb trilayers, and were produced at SEEQC in Elmsford (New York, NY, USA). The experiments were performed on two sets of chips with, respectively, 50 A/cm^2^ and 100 A/cm^2^ current density.

**Figure 2:**
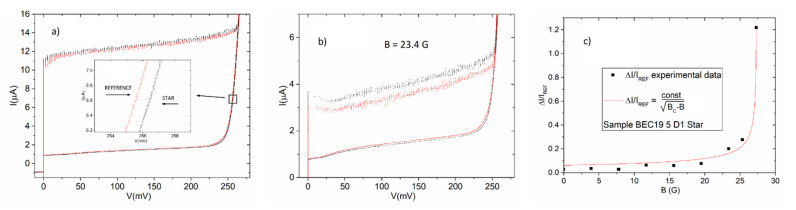
(**a**) The current-voltage characteristics of an array embedded in a star structure (black) and that of a reference array (red). The inset shows a zoom of the region in the square where we can see that the gap of each junction of the embedded array advances with respect to those of the reference array; (**b**) The characteristics of Figure 2a after the application of an applied magnetic field of 23.4 G: we see that the difference between of the currents of the embedded array (black) and that of the reference array (red) increases; (**c**) The dependence of the ratio between average currents of embedded array and reference array: increasing the magnetic field the ratio “diverges” following a typical functional dependence of phase transitions (for explanation see text).

In Figure 2a, we show the current voltage characteristics, respectively, of a 100 A/cm^2^ chip array embedded in the graph structure (black) and of the “reference” array (red) having the same geometrical shape but isolated. The two arrays are both formed by series arrays of 100 junctions and represent the overall connection of two series arrays located along two aligned rays of the star. We can see in the inset that for the embedded array the gap-sum voltage is higher by about 1 mV. This result is a consequence of the fact that each junction of the embedded array has a gap voltage slightly higher (roughly 10 μV) than that of the reference array. It is worth mentioning that a recent theoretical investigation has demonstrated that a gap (and transition temperature) increase can be expected in star-shaped arrays of superconductive islands connected by Josephson potentials [22].

In [18], a condition was explicitly expressed for the coupling energy between the islands in order to observe the theoretically predicted phenomena: Josephson coupling energy should be of the order of *k_B_T* (*k_B_* = 1.38 × 10^−23^ J/K is the Boltzmann constant). Now, for *T* = 4.2 K (and below) the zero bias Josephson energy (Φ_0_I_c_/2π) (here Φ_0_ = 2.07 × 10^−15^ Wb is the flux quantum) [23,24] is much larger than the thermal energy and no phenomena should be observed. As pointed out in [21], observations of the theoretical effects are possible when the height of the Josephson coupling potential (washboard-like shape) becomes very shallow (both in reference arrays and in embedded arrays, naturally) as we approach the maximum Josephson current by applying an external dc-bias current in order to trace current-voltage characteristics.

However, while in the dc-biased array that is being probed the coupling potential is shallow, in the dc-unbiased arrays the potential is high, meaning that the condition of the Josephson energy being of the same order as the thermal energy is not satisfied over the whole 2-d array. The condition of having a Josephson energy comparable with thermal fluctuations can either be obtained by fabricating junctions that have critical currents of the order of hundreds of nanoampères, or by lowering the Josephson current of all the junctions of the arrays by an external magnetic field.

We chose the second of the two possibilities applying a uniform external magnetic field to the array. We found that the differences in current amplitude observed between the Josephson currents of the embedded arrays and those of the reference arrays (we define this ΔI) have a dependence on the applied external field. In Figure 2a, we see that the critical currents of the embedded array are slightly larger than those of the reference arrays. Decreasing the Josephson energy by the application of an external magnetic field, the difference between the currents of the embedded array and those of the reference array becomes more pronounced, as we see in the inset of Figure 2b for a field of 23.4 G. In Figure 2c, we report the dependence of the difference between the Josephson currents of the embedded array and that of the reference array (ΔI) as a function of the applied magnetic field (this difference is normalized for each point magnetic field to the critical current of the reference array at the given field). The differences between embedded and reference arrays were measured for each field producing an average of the values of all the Josephson currents of the arrays. The line between the experimental data is the dependency $\frac{\Delta I}{I_{REF}}=\frac{const}{\sqrt{B_{c}-B}}$ which is a typical functional behavior associated to superconductive parameters, such as, for example, the dependence of London penetration depth and coherence length on temperature, near the superconducting transition [7,23].

Figure 2c shows what happens when the magnetic field is increased and the height of the Josephson currents becomes closer to the value for which the zero-bias Josephson energy equals *k_B_T* which, for a temperature of 4 K, is equal to 175 nA. Indeed, for the value of the field B = 27 G the Josephson junctions of the reference attain a critical current *I_C_* lower than 200 nA. At this point, all the junctions of star array (not only those current-biased) can participate to give rise to the theoretically predicted macroscopic quantum state [13] and the carrier flow through specific rays of the star can increase as a consequence. We must note that this is not in contradiction with the fact that gap enhancement is observed over all of the junctions of the arrays since the Josephson current is related to the gap through the Ambegaokar–Baratoff equation [24,25], based on Bardeen–Cooper–Schrieffer (BCS) theory; we also remark, however, that the observed Josephson current increases can be substantially higher than what one would expect from the corresponding gap increases, based on the BCS prediction.

**Figure 3:**
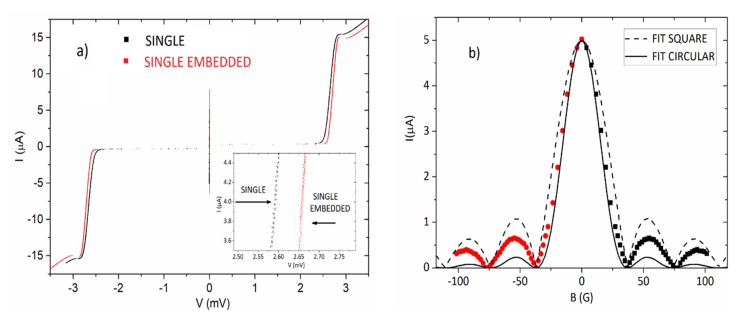
(**a**) Comparison between the current-voltage characteristics of two single junctions: one which is part of a finger array on a double comb array (black) and another which is isolated. We can clearly see in the zoom of the area (inset) that the embedded junction has a 60 μV higher gap at a current of 4 μA; (**b**) Diffraction pattern of the isolated junction: beside evidencing a “circular junction” tendency, likely due to fabrication process rounding in the corners, the pattern is very regular. We show both the ideal Fraunhofer pattern (continuous line) and the circular junction pattern (dashed line): the behavior of our sample is in between the two. We used red circles for negative fields and the black squares for positive fields.

Let us step now to the results obtained on a double comb array chip with a 50 A/cm^2^ current density; the layout of the array is shown in Figure 1b. Experimental data for this kind of topology were recently reported [26] and these clearly show increased superconductive gaps in the islands embedded in the comb structure, both for the “finger” and for the “backbone” arrays. In the present design, see Figure 1b, we included the possibility to contact directly a single junction belonging to the last “finger” on the right of the comb array; the junction is located 11 islands away from the backbone. Having the possibility to measure this individual junction, we compared its features with those of a single isolated junction (not embedded in the array). The comparison of the current-voltage characteristics of these two junctions is shown in Figure 3a: from the data available in this figure we recorded that the gap of the embedded junction was 2.66 mV, while that of the isolated junction was 2.6 mV. In Figure 3b, we also report the diffraction pattern of the isolated junction showing that the pattern is in between that of an ideal Fraunhofer pattern for a rectangular junction and that of a circular junction [24], an effect likely due to some “rounding” effects on the corners, caused by the fabrication procedure of the chips.

**Figure 4:**
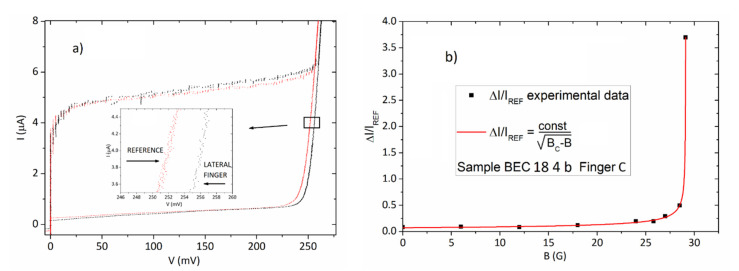
(**a**) The gap-sum array of an embedded “lateral” finger array and the reference array (geometrically equivalent) placed on the same chip; (**b**) The dependence for the normalized increase of average Josephson currents on an embedded central finger array (Finger C) with respect to the reference array as a function of the external magnetic field.

For the current-voltage characteristics shown in Figure 3a, the increase in the gap of the embedded junction, measured at 4 μA, was 65 μV. In Figure 4a, we show the current-voltage characteristics of a “lateral” finger array and that of the reference array. We can see that the gap increase observed for a lateral finger array, consisting of 100 junctions, measured at 4 μA, was 5 mV above the gap voltage sum of the reference array, meaning that the gap increase for a single junction observed in Figure 3a was present over all of the junctions. We must specify that an overall gap sum giving a difference with the reference array of 10.66 mV, was also observed at a current of 4 μA for a central finger array. We believe that this difference (roughly a factor 2) in gap increase between central and border arrays depends on finite size effect since the arrays situated at the end of the structure have gap increases lower than those located deep inside the graph structures.

In Figure 4b, we show the fitting of the dependence on the external magnetic field of the difference between the average Josephson currents of the central finger array and its reference array. Even in this case we see that, when decreasing the Josephson coupling, the difference between embedded and reference structures increases and a “phase-transition” functional dependence fits very well with the experimental data.

It is worth noting that the “isolated” single junction had a higher Josephson current than that of the embedded junction. This effect can be expected if the boson distribution follows that predicted in [13] for a finger array. Thus, in the phenomenon that we observed, the increase of Josephson currents is not linearly related to the enhancement of the gap as would be predicted by an overall increase in the density of states on the islands. Moreover, the “excess” gap is uniform over all of the junctions of the embedded structure, but can reach, in the best cases, values in the order of 4% of the gap of bare junctions, while the Josephson currents might have non uniformities that can reach values in the order of 15% [19,26].

## 3. Conclusions

Since the first reported evidence of anomalies in graph-shaped arrays of Josephson junctions [18], relevant steps forward in the understanding of the observed phenomena have been accomplished. This work has demonstrated that in the graph arrays, non uniform distribution of charge carriers along specific directions, as predicted by theoretical models, might exist: along the preferential directions of the network arrays where the bosons tend to accumulate, or decrease, the measured Josephson currents are larger, or smaller. It has been demonstrated that the reason for which the non uniformities in Josephson current distributions can be observed is due to the specific probing technique that we used: tracing current-voltage characteristics of the arrays implies switching to a voltage state close to the maximum of the Josephson current where the height of the Josephson washboard potential becomes very low and can be of the order of thermal fluctuations [21,24]. It has also been reported, however, that the anomalies of the current distribution can be so strong that they generate current levels close to the normal resistance level of the junctions [21].

We conclude that the comparison of the current-voltage characteristic of the arrays embedded in the graph structures with those of arrays having exactly the same geometry but not embedded in the structures (reference arrays), clearly shows that the observed effects have nothing to do with the specific geometry.

Theoretical analyses have reported on the interest of the structures that we have investigated from the fundamental mathematic point of view [14,27,28,29], and other publications [22,30,31,32] have demonstrated the interest from different perspectives for the type of systems we have investigated. It is worth pointing out that the phenomena that we observe are not just determining an increase of currents along specific directions of the graphs when the superconductors are in the condensed state: our results point toward the existence of new topological states in which fundamental properties of the superconducting state, such as gap and transition temperature, can be conditioned. We have shown that the amount of conditioning depends on the type of graph and, along a given “topological” direction, the increase in the gap is uniform over all of the islands: proof of this effect for the rays of a star array has been reported herein. We have reported an observation, to be further developed, of the effects that finite size could have on double-comb structures; the relevance of these type of effects has also been investigated for discrete Josephson structures in the framework of coherent oscillations and radiation generation [33].

## Data Availability

Not applicable.
